# UAV-Based Thermal, RGB Imaging and Gene Expression Analysis Allowed Detection of Fusarium Head Blight and Gave New Insights Into the Physiological Responses to the Disease in Durum Wheat

**DOI:** 10.3389/fpls.2021.628575

**Published:** 2021-04-01

**Authors:** Sara Francesconi, Antoine Harfouche, Mauro Maesano, Giorgio Mariano Balestra

**Affiliations:** ^1^Department of Agriculture and Forest Sciences (DAFNE), University of Tuscia, Viterbo, Italy; ^2^Department for Innovation in Biological, Agro-Food and Forest Systems (DIBAF), University of Tuscia, Viterbo, Italy

**Keywords:** Fusarium head blight, durum wheat, high-throughput plant phenotyping, thermal imaging, RGB imaging, gene expression, early disease detection

## Abstract

Wheat is one of the world’s most economically important cereal crop, grown on 220 million hectares. Fusarium head blight (FHB) disease is considered a major threat to durum (*Triticum turgidum* subsp. *durum* (Desfontaines) Husnache) and bread wheat (*T. aestivum* L.) cultivars and is mainly managed by the application of fungicides at anthesis. However, fungicides are applied when FHB symptoms are clearly visible and the spikes are almost entirely bleached (% of diseased spikelets > 80%), by when it is too late to control FHB disease. For this reason, farmers often react by performing repeated fungicide treatments that, however, due to the advanced state of the infection, cause a waste of money and pose significant risks to the environment and non-target organisms. In the present study, we used unmanned aerial vehicle (UAV)-based thermal infrared (TIR) and red-green-blue (RGB) imaging for FHB detection in *T. turgidum* (cv. Marco Aurelio) under natural field conditions. TIR and RGB data coupled with ground-based measurements such as spike’s temperature, photosynthetic efficiency and molecular identification of FHB pathogens, detected FHB at anthesis half-way (Zadoks stage 65, ZS 65), when the percentage (%) of diseased spikelets ranged between 20% and 60%. Moreover, in greenhouse experiments the transcripts of the key genes involved in stomatal closure were mostly up-regulated in *F. graminearum*-inoculated plants, demonstrating that the physiological mechanism behind the spike’s temperature increase and photosynthetic efficiency decrease could be attributed to the closure of the guard cells in response to *F. graminearum*. In addition, preliminary analysis revealed that there is differential regulation of genes between drought-stressed and *F. graminearum*-inoculated plants, suggesting that there might be a possibility to discriminate between water stress and FHB infection. This study shows the potential of UAV-based TIR and RGB imaging for field phenotyping of wheat and other cereal crop species in response to environmental stresses. This is anticipated to have enormous promise for the detection of FHB disease and tremendous implications for optimizing the application of fungicides, since global food crop demand is to be met with minimal environmental impacts.

## Introduction

Wheat is one of the most cultivated crops worldwide, grown on 220 million hectares and its annual production is estimated to account for more than 700 million tons, providing more than 20% of total human food calories (Khan et al., 2020; Ma et al., 2020). Modern wheat cultivars derive from two species: bread wheat (*Triticum aestivum* L.) and durum-type wheat (*T. turgidum* subsp. *durum* (Desfontaines) Husnache) used for pasta and low-rising bread (Peng et al., 2011; Feldman and Levy, 2012).

The current food demand will double with a projected population of 9 billion in 2050. In response, farmers must grow more on their lands through sustainable intensification of agriculture, an approach to increase yield production without having negative effects on the environment or expanding the agricultural footprint (Giller et al., 2015).

Wheat production is challenged by several abiotic and biotic stresses. Among the plant fungal diseases, Fusarium head blight (FHB) is one of the most destructive diseases leading to 10–70% of yield loss during the epidemic years (McMullen et al., 2012). FHB is caused by the *Fusarium graminearum* species complex (FGSC), which embraces 16 different species. It is considered the most devastating wheat disease due to the lack of resistant cultivars, the significant yield loss and grain quality reduction, and the health risks associated with the contamination of crops with mycotoxin such as deoxynivalenol (DON) and zearalenone (ZEA), produced during the infection progress (Yang et al., 2013; Dweba et al., 2017). The spectrum of *Fusarium* spp. causing FHB on wheat varies at the regional level, depending on weather conditions. Fungal growth is favored by high temperatures and humidity during the growing season. *F. graminearum* Schwabe is the predominant species that causes FHB in many countries, in Asia, North America, South America, and Europe (Dweba et al., 2017; Khan et al., 2020). In field conditions, the inoculum occurs primarily from plant residues and soils while the dissemination of the ascospores and conidia is mainly promoted by water splash and wind. Anthesis is the most susceptible stage to infection. With a warm and humid climate at this stage, airborne spores proliferate and spread intracellularly into the rachial nodes and through the rachis until FHB symptoms are clearly visible. The symptoms include necrosis, premature bleaching of spikes, and shriveled kernels that negatively affect photosynthesis. At favorable conditions, FHB develops rapidly within 3–6 days after the infection. Given the current global warming associated with increased temperatures, major epidemics of FHB are occurring. Thus, proper cultivation schemes and field management are essential to alleviate its threat (Bai and Shaner, 2004; Vaughan et al., 2016; Ma et al., 2020; Rod et al., 2020).

Since 1995, *Fusarium* spp. infect wheat in Italy at various incidence (percentage of spikes showing symptoms) and severity (percentage of areas infected in spikes) depending on the year, the region, and the wheat cultivar involved (Pancaldi et al., 2010). Its incidence and severity are closely related to the amounts of precipitation during wheat anthesis, increasing from the South to the North of Italy, being mostly reported in the Northern-Central regions (Shah et al., 2005; Infantino et al., 2012). Between the two major species, *T. turgidum* is the most widely grown in Italy, but is also more susceptible to FHB (Oliver et al., 2008). Consequently, being mostly used for human consumption, the risk of mycotoxin-contaminated grain entering the food chain is a major concern (Boutigny et al., 2008).

Chemical control of FHB using appropriate effective fungicides and correct application methods and timing can reduce the severity of the disease (Blandino et al., 2012). However, no fully effective FHB fungicide is available (Haidukowski et al., 2012), and the application window is very narrow, spanning just a few days around anthesis (Mesterházy et al., 2003). Recently, an organic strategy to reduce FHB incidence and severity was proposed, but now it needs to be confirmed on wide areas (Francesconi et al., 2020). For these reasons, early detection and control of FHB is a key factor to gain maximum protection of yield (Mahlein et al., 2019) from fungal spread and mycotoxin accumulation; in fact, there is a strong evidence base in the research literature that a prompt and early application of fungicides instead of applying fungicides at late stages of infection drastically reduce the FHB spread and DON accumulation inside the grains, instead of applying fungicides at late stage of infection (Freije and Wise, 2015; Feksa et al., 2019; Bolanos-Carriel et al., 2020).

Recent advances in biological and bioanalytical research enabled genome-scale capturing of biological processes at the molecular level in plants (Weckwerth, 2011). Though transcripts evaluation is fundamental to understand plant responses to pathogens, these techniques are labor-intensive, time-consuming, destructive and slow-down the acquisition of phenotype parameters related to the gene responses, contributing to the phenotyping bottleneck (Furbank and Tester, 2011). Molecular data obtained in greenhouse or field trials combined with phenotypic and environmental data discloses a wealth of information that can be used to improve field management (Alexandersson et al., 2014).

For plant-pathologists, coupling transcriptomics and phenomics to agricultural practices is likely to have a large impact on the understanding of induced plant defenses and pathogen spread. In fact, phenomics and transcripts analysis can reveal important physiological changes in plants in response to pathogens which can help detecting infections before the appearance of their visible symptoms. Hence, these associated techniques have the potential to be a powerful weapon that optimizes fungicide spraying regimes for plant pathogen management (Alexandersson et al., 2014).

To relieve the phenotyping bottleneck, phenotypic traits should be turned into quantifiable, objective, and consistent measures (Ludovisi et al., 2017). Automated and high-throughput phenotyping (HTP) provides measurements that can track the development of a plant through its life stages and its interaction with the environment, establishing methodologies to detect crucial physiological traits and identify effective genotype-phenotype relationships (Goggin et al., 2015). These methods speed up the phenotyping process and maximize the number of studied plants per experiment (Goggin et al., 2015). Furthermore, they enable automated, non-destructive, and non-invasive screening of large populations and high dimensionality data (Li et al., 2014; Fahlgren et al., 2015).

With regards to FHB, *Fusarium* spp. infects spikelets within wheat spikes, decreasing stomatal conductance, and thus, affecting the chlorophyll and water content (Yang et al., 2016; Francesconi and Balestra, 2020). This allows its early detection by color imaging and by measuring the temperature of spikes and photosynthetic efficiency of plants using thermometers and fluorometers, respectively (Cambaza et al., 2019; Mahlein et al., 2019).

Red-green-blue (RGB) cameras are designed to emulate human vision by sensing light in the visible range of the electromagnetic spectrum (wavelengths from 390 to 700 nm). In this range, the reflectance is predominantly influenced by plant pigments (e.g., chlorophyll) (Mahlein, 2016). This allows the calculation of different vegetation indices (VIs) by computing the reflectance of a certain band of the green and red zone of the electromagnetic spectrum (Barbosa et al., 2019; Brunori et al., 2020). Photosynthetic response of green vegetation to incident light is the basis of VIs where healthy plants exhibit low red reflectance due to absorption of red light by chlorophyll resulting in a high index value, whereas unhealthy, stressed plants with reduced chlorophyll pigment display a low index value (Khan et al., 2018).Therefore, RGB image analysis can serve as an important tool that detects physiological changes in plants caused by pathogen infections (Simko et al., 2016). On the other hand, thermal infrared (TIR) sensors capture images containing information about the energy emitted from body surfaces, such as plant canopies. Plant pathogens affect the loss of water in plants regulated by stomata (Fang and Ramasamy, 2015), altering plant temperature where high values indicate areas with closed stomata (Oerke et al., 2006; Chaerle et al., 2007). Thermography can serve as a tool to measure these changes toward an early detection of plant infections, ideally before symptoms appear (Al Masri et al., 2017).

Unmanned aerial vehicles (UAVs) equipped with cameras and sensors have become advanced field phenomics tools that provide data with high spatial and temporal resolution to bridge the gap between time consuming ground-based measurements and satellite observations (Xie and Yang, 2020; Pineda et al., 2021). UAVs allow rapid and non-destructive measurements and offer much quicker turnaround times than satellites at competitive costs (Ludovisi et al., 2017).

The application of UAV-based imaging techniques has been broadening in several areas of agricultural sciences thanks to their ability to analyze plant temperature and color discrepancies between distinct biological samples (Padmavathi and Thangadurai, 2016; Cambaza et al., 2019). Many recent studies have exploited UAV-based sensors to monitor, detect and phenotype plant stresses in forestry (Sagan et al., 2019), as well as to estimate leaf nitrogen concentration (Lu et al., 2021), water and nitrogen use efficiencies (Yang et al., 2020), and salinity stress (Johansen et al., 2019) in different crops. Plant pathologists are recently also benefiting from the application of UAV-based sensors; in fact, UAV multispectral and hyperspectral imaging have been used to detect *Xylella fastidiosa* (Castrignanò et al., 2021) and FHB (Liu et al., 2020), respectively. Moreover, these techniques are relatively easy to use and are becoming cheaper (Dammer et al., 2011; Cambaza et al., 2019). Their exploitation to monitor FHB can contribute significantly to secure the cereal production systems (Mahlein et al., 2019). In light of these advantages, UAV-mounted cameras and sensors are expected to enable new applications in field-based phenotyping of plant stress traits in large populations rapidly, precisely and accurately (Berni et al., 2009; White et al., 2012; Yang et al., 2017).

In this study, we exploited plant physiological and molecular changes in response to *Fusarium* spp. infection to detect FHB in *T. turgidum* (cv. Marco Aurelio) fields through UAV-based TIR and RGB imaging. Particularly, (i) two VIs as well as spike temperature were calculated using RGB and TIR images, from both uninfected and infected areas and were correlated to photosynthesis perturbation caused by the infection. Additionally, (ii) transcripts of key genes involved in stomatal conductance regulation were investigated in greenhouse experiments, to explore the genotypic changes behind the observed phenotypic perturbations (increase in spike temperature and decrease in photosynthetic efficiency). Furthermore, (iii) transcripts of *F. graminearum* inoculated plants were compared to those obtained from drought-stressed ones in order to investigate a differential response between these two types of stresses.

## Materials and Methods

### Plant Material and Experimental Design of the Field Experiments

**FIGURE 1:**
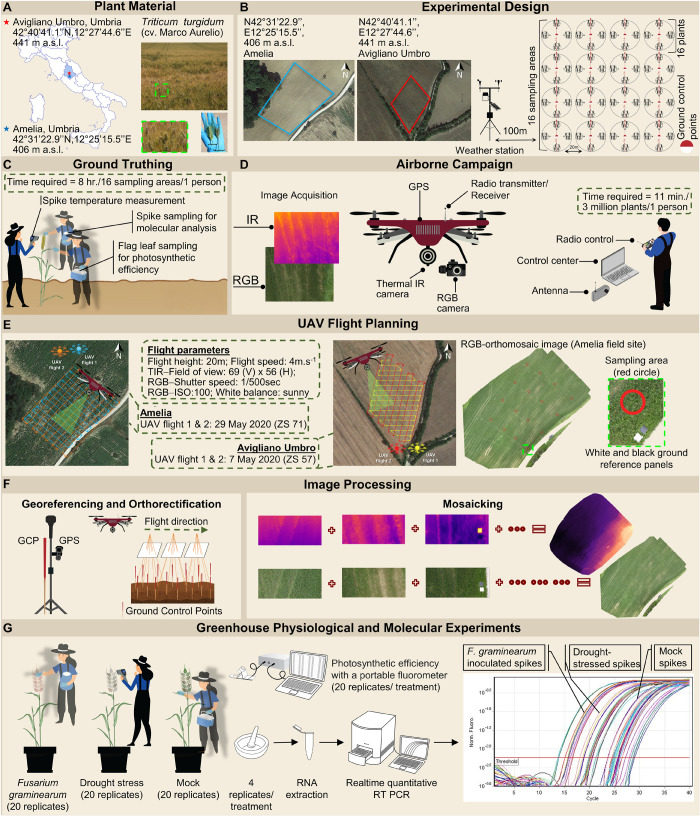
Workflow of the high-throughput field phenotyping methodology and the greenhouse experiment. **(A)** Plant material includes *Triticum turgidum* (cv. Marco Aurelio). Two fields were established in Amelia (Central Italy, 42°31′22.9′′N, 12°25′15.5′′E, Umbria Region, 406 m above sea level) and Avigliano Umbro (Central Italy, 42°40′41.1′′N, 12°27′44.6′′E, Umbria Region, 441 m a.s.l) to host the *T. turgidum* plants. Sampled spikes show the symptoms of a Fusarium head blight (FHB)-infected plant, compared to an uninfected plant. **(B)** An experimental plot was allocated within each field, and a 20 m grid was identified by positioning sixteen ground control points (GCPs) to be used for georeferencing. Around each of the GCPs, a circumference of 1 m of diameter was individuated and denominated as a sampling area. Weather data were recorded daily by two meteorological stations installed at 100 m distance from each field. **(C)** Within each sampling area, the spike temperature of sixteen randomly selected spikes was measured, and their flag leaves were sampled to measure their photosynthetic efficiency. Finally, the spikes were also sampled to distinguish FHB positive (FHB+) ones from FHB negative (FHB-) ones by molecular identification of *Fusarium* spp. **(D)** An unmanned DJI Matrice 600 hexacopter was equipped with Zenmuse X5 red-green-blue (RGB) and Zenmuse XT thermal infrared (TIR) sensors. The unmanned aerial vehicle (UAV) campaign allowed capturing RGB and TIR images of both experimental plots. **(E)** The flight missions were planned using Ground Station Pro app (DJI GS Pro, China). The UAV was flown in the autonomous mode at a nominal speed of 4 m/s, running parallel to plant rows to ensure complete coverage with good overlaps. **(F)** Image orthorectification, georeferencing, and mosaicking were performed using 16 GCPs captured with a global positioning system (GPS). **(G)** The greenhouse physiological and molecular experiments where plants were subjected to three different treatments: (i) drought stress treatment where plants did not receive water from Zadoks stage (ZS) 51 to 65; (ii) artificial inoculation treatment where spikes were uniformly spray-inoculated at ZS 65 with a suspension of 1 × 10^5^ conidia/mL; and (iii) mock treatment where spikes were uniformly sprayed with a suspension of Tween-20 0.05% resuspended in sterile distilled water at ZS 65. Spikes temperature and photosynthetic efficiency of plants were recorded at 24, 48, and 72 hours post inoculation (hpi) from three independent experiments, each experiment consisting of 20 spikes for each treatment. Four spikes were sampled for each treatment, and were ground with mortar and pestle in liquid nitrogen until a fine powder was obtained, from which, RNA was extracted. Finally, quantitative real-time polymerase chain reaction (RT-qPCR) was performed to investigate transcripts of key genes involved in stomatal conductance regulation; transcripts of *F. graminearum*-inoculated plants were compared to those obtained from drought-stressed ones in order to investigate a differential response between these two types of stresses.

The FHB-susceptible *T. turgidum* cv. Marco Aurelio was the cultivar of interest in the present work for both field and greenhouse experiments. This genotype, grown in Central and South Italy, is extensively used for pasta production and it is characterized by excellent protein content and high productivity, thus it is of high economic importance. The experimental fields were located in Amelia (Central Italy, 42°31′22.9′′N, 12°25′15.5′′E, Umbria Region) and Avigliano Umbro (Central Italy, 42°40′41.1′′N, 12°27′44.6′′E, Umbria Region). The *T. turgidum* field experiments were carried out during two consecutive years (2019 and 2020), controlled and drip-irrigated with a nutrient solution containing nitrogen, potassium, phosphorus, and small amounts of other compounds. Therefore, abiotic stresses (nutrient deficiencies and drought stress), diseases, or pests, which cause the same symptoms as FHB, were significantly minimized. On the other hand, fungicides for FHB management (tebuconazole and azoxystrobin) were not applied in order to favor the FHB natural infection. The fields were investigated periodically (one time per day) by experts and farmers to prevent other diseases, pests, or abiotic stress. An experimental plot was allocated within each field, and individuated by a 20 m grid by positioning 16 ground control points (GCPs) to be used for georeferencing. During March, April, and May 2019 and 2020 weather data (minimum, maximum and average temperature, and precipitation) were recorded daily by two meteorological stations installed at 100 m distance from each field to monitor the climate factors influencing the establishment of FHB infection. Historical weather data (from 2010 to 2018) were obtained from the Hydrographic service of Umbria Region^1^ to monitor the climatic trend of the last 11 years and to compare minimum, average and maximum temperature values (°C) and average precipitations (mm) recorded during the 9-year seasonal average (2010–2018), 2019, and 2020. The historical weather data were collected from two meteorological stations located in Amelia (42°33′25.0′′N, 12°25′01.0′′E) and Avigliano Umbro (42°40′39.0′′N, 12°26′13.0′′E). The experimental design for the field experiments is illustrated in Figures 1A,B.

### UAV Campaigns

An unmanned DJI Matrice 600 multi-copter (DJI, China) equipped with a Zenmuse X5 RGB camera (DJI, China) and a Zenmuse XT TIR camera (DJI, China) has been used in this study (Supplementary Figure 1). DJI Matrice 600 is a hexa-copter (110 cm diagonal size) with a highly resistant carbon fiber frame, offering a 15 kg take-off weight. Its maximum transmission distance is 5 km and its maximum flight time is 40 min (Supplementary Figure 1).

The Zenmuse XT was equipped with a 9 mm f1.4 lens. Its thermal sensitivity is less than 50 milliKelvins and the camera enables measurements in the range −25°C to +135°C. The image sensor is a focal plane array (FPA) based on uncooled microbolometers with a spectral band response in the range of 7.5 to 13 μm. The camera field of view is equal to 69° (horizontally) × 56° (vertically), its resolution to 640 × 512 pixels at 30 frames per second (fps), and its spatial resolution to 1.889 milliradians (mrad). The camera captures images at an acquisition frequency of 30 Hz. The camera was radiometrically calibrated to further increase temperature measurement precision by setting external parameters during the flight planning such as air temperature and flat field correction (FFC). This is an offset calibration usually performed at power up, when the camera changes temperature, and periodically during the operation. This calibration compensates for certain errors that build up during the camera operation. During the data acquisition phase, the auto gain mode parameter was applied; the camera automatically selected the optimal gain mode according to the temperature range of the image (Deery et al., 2016; Gómez-Candón et al., 2016; Ludovisi et al., 2017) and pictures were stored as 14-bit digital raw images.

The Zenmuse X5 is a 16-megapixel RGB camera equipped with an M4/3 sensor enabling it to capture detailed images at a resolution of 4608 × 3456 pixels and an ISO range of 100–25600.

The Zenmuse X5 and XT cameras were mounted on a highly reliable gyrostabilized 3-axis gimbal (DJI, China) that automatically stabilizes them in flight. The gimbal constantly communicates with the UAV, and quickly compensates for every minor movement with a precision accuracy of 0.02°.

The 16 GCPs were used to georeference RGB images. For calibration of thermal images, four 60 × 60 cm ground reference panels (GRPs) consisting of two plastic panels covered by black vinyl tape and two white Teflon^®^ were positioned along the borders of each of the two study areas. A real-time kinematic (RTK) global navigation satellite system (GNSS) CS10 model (Leica Geosystems, Switzerland) with an accuracy of 1 mm was used for capturing GCPs and GRPs locations.

UAV campaigns were conducted at the following phenological stages: (i) three-quarters of inflorescence emerged (Zadoks stage 57, ZS 57) (Zadoks et al., 1974) on 6 May 2019 and 7 May 2020; (ii) anthesis half-way (ZS 65) on 17 May 2019 and 18 May 2020; and (iii) kernel watery ripe (ZS 71) on 28 May 2019 and 29 May 2020. Two flights were performed during each campaign. The flight missions have been planned using Ground Station Pro app (DJI GS Pro, China). Each flight lasted 11 min covering an area of 1 ha at a nominal speed of 4 m/s and an altitude of 20 m, during which both cameras acquired nadiral images with 90% frontal and side overlap. To ensure similar solar illumination angles flights were performed between 11:00 and 12:00 local time under stable cloudless and low-wind conditions (Figures 1D,E).

### Ground Measurements

For each of the 16 GCPs, a circumference of 1 m of diameter was individuated and denominated as sampling area. During the UAV campaigns, we manually measured the spike temperature of sixteen randomly selected plants in each of the 16 sampling areas using a portable infrared thermometer (Fluke 568, Fluke Corporation, United States) with an accuracy of ± 1% or ± 1.0°C (whichever is greater), positioned at 10 cm distance from the spike. At the same time, the FHB severity was calculated by counting the number of diseased spikelets and the total number of spikelets for each of the sixteen plants. Furthermore, the flag leaves of the selected plants were sampled and dark-adapted for 1 h before measuring their photosynthetic efficiency, by quantifying F_v_/F_m_ reflecting the potential quantum efficiency of photosystem II (Maxwell and Johnson, 2000), with a portable fluorometer (V2.00f PAM 2000, Heinz Walz GmbH, Germany). Moreover, the spikes were also sampled to distinguish FHB positive (FHB+) from FHB negative (FHB-) areas by molecular identification of *Fusarium* spp. (Figure 1C).

### Molecular Diagnostics of *Fusarium* Pathogens

Spikes were stored in a portable fridge immediately after sampling. Afterward, different tissues (palea, lemma, glume, rachis, and kernel when present) from each collected spike were plated on Petri dishes containing potato dextrose agar (PDA) and incubated at 21°C for 72 h. The different morphotypes were subsequentially isolated and cultured on PDA for 1 week. After each morphotype filled the Petri dish, the produced mycelium was gently scraped with a glass rod. 100 mg of mycelium were grinded and DNA was extracted using 80 μL of extraction buffer composed of Tris 100 mM, EDTA 50 mM and NaCl 500 mM; after that, 32 μL of SDS 10% (w/v) were added and the samples were incubated at 65°C for 10 min. 27 μL of potassium acetate 5 M were added and the samples were placed on ice for 20 min, then centrifuged for 20 min at 13.000 rpm. The supernatant was recovered and 80 μL of cold (−20°C) isopropanol were added to each sample; then, the samples were placed on ice for 10 min and centrifuged for 5 min at 13.000 rpm. The supernatant was discarded and 150 μL of cold (−20°C) ethanol (70% v/v) were added to the samples. Samples were centrifuged for 3 min at 13.000 rpm, the supernatant was discarded and the DNA was resuspended in 20 μL of DNase and RNase-free sterile distilled water and stored at −20°C (D’Ovidio and Porceddu, 1996). Total DNA was quantified with Qubit^TM^ fluorometer 1.01 (Invitrogen, United States) using the Qubit^TM^ dsDNA BR Assay Kit (Thermo Fisher Scientific, United States) and diluted to 10 ng/μL. The molecular identification of *Fusarium* spp. was performed by amplifying the translational elongation factor 1-α (*TEF*) sequence using the primer pair EF1_F 5′-ATGGGTAAGGAGGACAAGAC-3′/EF2_R 5′-GGAAGTACCAGTGATCATGTT-3′ designed to identify the FHB complex spp. (Geiser et al., 2004). The polymerase chain reaction (PCR) was performed following the instructions of GoTaq^®^ Green Master Mix (Promega, United States) and prepared in a total volume of 10 μL. The amplification conditions consisted of: (i) an initial denaturation step of 2 min at 95°C; (ii) 35 cycles of 30 s denaturation at 95°C; (iii) 40 s of annealing at 53°C; (iv) 60 s of elongation at 72°C; and (v) a final elongation step of 5 min at 72°C. The amplicon unicity was visualized on 1.5% agarose gel and sequenced by Sanger sequencing (Eurofins Genomics, Germany). The resulted sequences were submitted to BLASTn^2^ in order to identify the corresponding *Fusarium* spp.

### RGB Image Processing and VIs Calculation

A total of 250 RGB images were acquired using the UAV-mounted Zenmuse X5 camera. The camera exposure mode was set to shutter priority (S) with a shutter speed fixed 1/500 s to ensure minimization of motion blur, ISO of 100 and the white balance to sunny mode.

Geometric camera calibration, orthorectification, and mosaicking of the captured images were performed using Pix4Dmapper (Pix4D, Switzerland) software, specifically designed to process UAV images using techniques rooted in both computer vision and photogrammetry to match conjugate points in overlapped images and to define their relative positions and orientations using bundle block adjustments (Bollard-Breen et al., 2015; Nishar et al., 2016; Figure 1F). The output of this step was the RGB orthomosaics of the study areas for each flight mission. No filtering process was applied to the images.

Finally, VIs from the orthomosaics were computed using quantum geographic information system (QGIS) software (version 3.4 Madeira - QGIS Development Team, Open Source Geospatial Foundation). The VIs carried out were: vegetative (VEG) (Hague et al., 2006) and green leaf index (GLI) (Louhaichi et al., 2001). VEG and GLI were calculated using the following formulas:

$$VEG=\frac{G}{R^{a*}B^{\left(1-a\right)}}\text{and}$$

$$GLI=\frac{\left(2^{*}G-R-B\right)}{\left(2^{*}G+R+B\right)}$$

where R, G, and B are the reflectance of red, green and blue channels, respectively, and *a* is equal to 0.667.

Inside each sampling area, leaves and spikes were not separated in the images, because the upper part of the fields was uniformly composed by spikes (Figures 1A,B), since wheat canopies were very dense.

### TIR Image Processing and Temperature Extraction

The UAV-mounted Zenmuse XT TIR camera captured 219 greyscale and georeferenced images. Thermal orthomosaics were generated for each flight, and the radiometric conversion was automatically performed using the Pix4Dmapper software (Figure 1F). The removal of bare soil pixels was not necessary, since the wheat planting were extremely dense, reducing the mixed pixel problem. FLIR Tools software (2020^©^ FLIR^®^ Systems, United States) was used to calibrate temperatures in TIR images using the temperature values of GRPs measured immediately after the UAV flights using a portable infrared thermometer (Fluke 568, Fluke Corporation, United States). Calibration checks were performed by comparing the GRPs-measured to the UAV-derived temperatures. No filtering process was applied to the images. The extraction of temperature values was performed for each of the 16 sampling areas. Inside each sampling area, leaves and spikes were not separated in the images, because the upper part of the fields was uniformly composed by spikes (Figures 1A,B), since wheat canopies were very dense.

### Plant Growth, Inoculation Conditions and Experimental Design of the Greenhouse Experiments

Greenhouse experiments were conducted in a glasshouse located in Viterbo (Central Italy, 42°25′35.8′′N, 12°04′49.3′′E, Lazio Region). The surface of the *T. turgidum* kernels was sterilized with sodium hypochlorite (0.5% v/v) for 20 min and rinsed twice with sterile distilled water for 5 min. The kernels were germinated in the dark on a paper, soaked in sterile distilled water for 15 days at 4°C to break dormancy, followed by 2 days at room temperature. The seedlings were transferred to 40 × 20 cm pots, filled with TYPical Brill soil (Brill, Germany) and grown at 16–20°C up to the boot stage (ZS 51), 20–24°C during heading and anthesis (ZS 53-69), and 24 –29°C up to maturity (ZS 71-99). The plants were fertilized using ammonium nitrate in the following proportions and at the following stages: 20% at sowing (ZS 00), 40% at tillering (ZS 20), and 40% at heading (ZS 49) (Francesconi et al., 2019). The highly virulent and mycotoxin-producing isolate of *F. graminearum* wild type (WT) 3824 (Tomassini et al., 2009) was cultured at 21°C on synthetic nutrient-poor agar (SNA) to obtain macroconidia (Mandalà et al., 2019). After 10 days on SNA, the conidia were scraped with a glass rod after pipetting 1 mL of sterile distilled water onto the surface of a Petri dish. The conidial suspension was recovered, and the concentration was adjusted to 1 × 10^5^ conidia/mL using a Thoma chamber (0.100 mm depth and 0.0025 mm^2^). The inoculum was prepared in sterile distilled water supplemented with 0.05% (v/v) of Tween-20. Plants were subjected to three different treatments: (i) drought stress treatment where plants did not receive water from ZS 51 to 65 (Zadoks et al., 1974; Tambussi et al., 2000); (ii) artificial inoculation treatment where spikes were uniformly spray-inoculated at ZS 65 with a suspension of 1 × 10^5^ conidia/mL; and (iii) mock treatment where spikes were uniformly sprayed with a suspension of Tween-20 0.05% resuspended in sterile distilled water at ZS 65. The spikes were covered with clear plastic bags for 24 h to maintain high humidity levels (>80%). Spikes subjected to drought and mock treatments were sampled after removing the plastic bags, while the inoculated spikes were sampled 24, 48 and 72 hours post inoculation (hpi) to investigate an early response to *F. graminearum*. Collected spikes were immediately stored in liquid nitrogen at −80°C until the extraction of RNA. FHB severity (%) was monitored in the greenhouse by counting the number of bleached spikelets and the total number of spikelets for each spike from 3 to 21 days post inoculation (dpi). In addition, spike temperature and photosynthetic efficiency were recorded at 24, 48, and 72 hpi. The experimental design for the greenhouse trial is illustrated in Figure 1G. Data were obtained from three independent experiments, each experiment consisting of 20 spikes for each treatment.

### RNA Extraction and cDNA Synthesis

Wheat spikes were ground with mortar and pestle in liquid nitrogen until a fine powder was obtained. The RNA was extracted from 100 mg of powder following the instructions provided by InviTrap^®^ Spin Plant RNA Mini Kit (Stratec Molecular GmbH, Germany), resuspended in RNase-free sterile distilled water, immediately poured onto ice and quantified with Qubit^TM^ fluorometer 1.01 (Invitrogen, United States) using the Qubit^TM^ RNA BR Assay Kit (Thermo Fisher Scientific, United States). To confirm the total quantity and integrity of the RNA, 5 μL of the extracted RNA sample was subjected to a 10-min thermal shock at −80°C, followed by 5 min at 65°C and run on 1.5% denaturing agarose gel. The synthesis of cDNA was performed using 500 ng of RNA following the instructions provided by Xpert cDNA Synthesis Supermix with a gDNA eraser (GriSP Research Solutions, Portugal) in a final volume of 20 μL. To ensure that the synthesis of the cDNA and the elimination of the gDNA had succeeded, a reverse transcription PCR (RT-PCR) of *T. aestivum* Actin (*TaACT*) (containing an intron in the amplified sequence) was performed following the instructions provided by GoTaq^®^ Green Master Mix (Promega, United States) in a total volume of 10 μL. The amplification conditions consisted of: (i) an initial denaturation step of 2 min at 95°C; (ii) 35 cycles of 30 s denaturation at 95°C; (iii) 40 s of annealing at 60°C; (iv) 30 s of elongation at 72°C; and (v) a final elongation step of 5 min at 72°C. The amplification run included a no-template control (NTC) and a genomic DNA (gDNA) control. The amplicons were visualized on 1.5% agarose gel.

### Gene Expression by Quantitative Real-Time PCR

Supplementary Table 1 shows the list of target genes, their functions, and the corresponding primer pairs used to perform RT quantitative PCR (RT-qPCR) (Francesconi and Balestra, 2020). Briefly, the primer pair for *T. aestivum* glyceraldeyde-3-phosphate dehydrogenase (*TaGAPDH*) amplification is from Jarošová and Kundu (2010), for *T. aestivum* pathogenesis related protein 1 (*TaPR1*) from Lu et al. (2006), for *TaACT* from Tundo et al. (2016), and *T. aestivum*β-tubulin2 (*TaTUB*) and *T. aestivum* ferredoxin-NADP(H)-oxidoreductase (*TaFNR*) from Tenea et al. (2011). The remaining primers are from Francesconi and Balestra (2020). The amplification efficiency (E) of RT-qPCR was determined for each primer pair as follows: five 1:10 serial dilutions (1:1-1:10000) were obtained for each cDNA and amplified in four replicates. E and coefficient of determination (*R*^2^) values were calculated by means of the slope of the standard curve obtained by plotting the fluorescence versus the serial dilution concentrations using the equation (Bustin et al., 2009)

$$E10^{\left(-\frac{1}{slope}\right)}-1$$

Reference genes with closest E values to target genes, highest *R*^2^, and lowest variability were selected for the quantification cycles (Cq). The relative expression levels of target genes were calculated on the basis of the Cq values of four independent biological replicates, each with four technical replicates, for each plant treatment by applying the equation (Bustin et al., 2009)

$$Relativeexpression2^{-△△Cq}$$

using *TaACT*, *TaTUB*, and *TaFNR* as reference genes and the mock treatment to normalize the relative expression levels. Relative expression levels of *TaPR1* and *TaGAPDH* were quantified as internal control of the progression of the infection (Muthukrishnan et al., 2001) and changes in photosynthesis (Zhang et al., 2013). The RT-qPCR was performed following the instructions provided by Rotor-Gene Q (Qiagen, Germany) and Xpert Fast SYBR (uni) MasterMix (GRiSP Research Solutions, Portugal), in a final volume of 10 μL. The amplification conditions consisted of: (i) an initial denaturation step of 3 min at 95°C; (ii) 40 cycles of 5 s denaturation at 95°C; (iii) 30 s of annealing at 60°C; and (iv) 20 s of elongation at 72°C. A final melt cycle (70–99°C) was performed to confirm the unicity of the amplicons. NTC controls were included and the amplification was considered negative when a value of Cq ≥ 38 was detected (Bustin et al., 2009).

### Photosynthetic-Related Parameters Measurements

Spike temperature and photosynthetic efficiency were measured for each plant treatment as performed during the field trial, described in section “Ground Measurements.” Photosynthetic-related parameters were measured for three independent replicates, each consisting of 20 individual spikes, for every treatment.

### Statistical Analyses

One-way analysis of variance (ANOVA) was performed to analyze FHB severity, ground-measurements (temperature and photosynthetic efficiency) of FHB+ and FHB- spikes during the UAV-campaigns, UAV-based VEG, GLI and temperature of FHB+ and FHB- sampling areas during the UAV-campaigns, gene expression values, and temperature and photosynthetic efficiency of drought stressed and inoculated plants during the greenhouse experiments. One level of significance (*p* < 0.01) was computed to assess the significance of the F values. A pairwise analysis was carried out using Tukey’s honest significant difference (HSD) test at 0.99 confidence level. Statistical analyses were performed using XLSTAT 2020.4 software (Addinsoft, France). Principal component analysis (PCA) was carried out to classify spike temperature, photosynthetic efficiency and VEG or GLI or UAV-based temperature values coming from FHB+ or FHB- areas and gene expression values, spike temperature and photosynthetic efficiency coming from drought stressed or *F. graminearum* inoculated plants. Heatmap was carried out by computing the *z*-score of the relative gene expression values. PCA and heatmap were computed by using ClustVis software (Tartu, Estonia)^3^ (Metsalu and Vilo, 2015).

## Results

### Weather Conditions Influencing the FHB Severity

**FIGURE 2:**
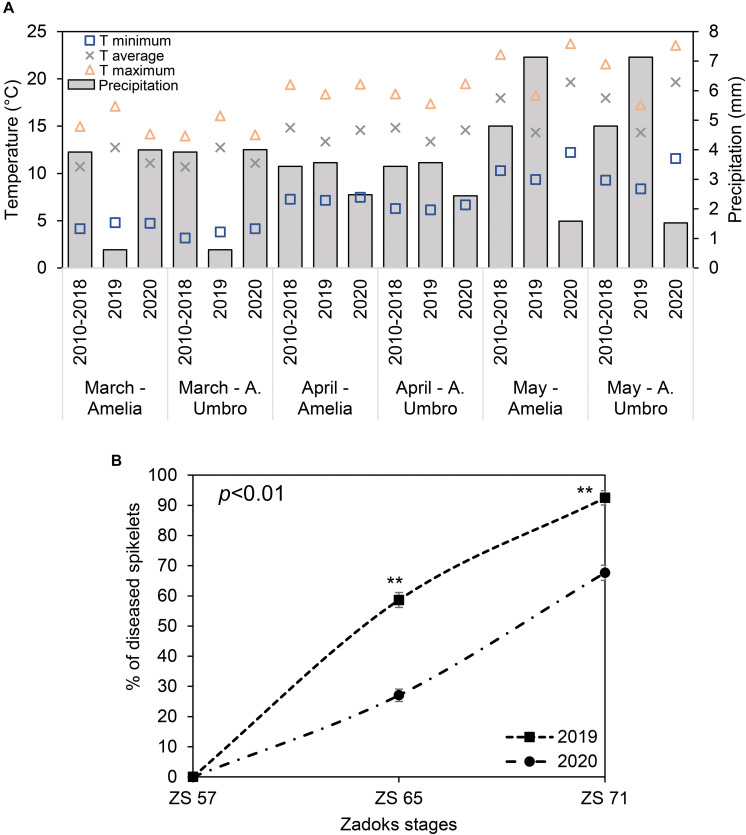
**(A)** Changes in maximum (yellow triangles), average (grey crosses) and minimum (blue squares) temperature (T), and precipitation (simple bar) of weather data recorded in 2019 in comparison with weather data recorded in 2020 and historical weather data from 2010 to 2018 for March, April, and May. The plotted values were obtained by averaging the mean daily values for each considered period. Weather data for 2019 and 2020 were recorded daily by two meteorological stations installed at 100 m distance from each of the experimental fields located in Amelia (Central Italy, 42°31′22.9′′N, 12°25′15.5′′E, Umbria Region) and Avigliano Umbro (Central Italy, 42°40′41.1′′N, 12°27′44.6′′E, Umbria Region). Historical weather data (2010–2018) were collected from the Hydrographic service of Umbria Region (https://annali.regione.umbria.it/). The historical weather data were collected from two meteorological stations located in Amelia (42°33′25.0′′N, 12°25′01.0′′E) and Avigliano Umbro (42°40′39.0′′N, 12°26′13.0′′E). **(B)** Severity percentage of Fusarium head blight (FHB) in *Triticum turgidum* (cv. Marco Aurelio) at Zadoks stage (ZS) 57, 65, and 71 in 2019 and 2020. Data represent averages and standard errors of 256 spikes (16 spikes for each of 16 visible targets). Asterisks (**) refer to the statistical analyses performed using one-way analysis of variance (ANOVA) with Tukey’s honest significant difference (HSD) post hoc test at 0.99 confidence level and *p* < 0.01.

Registered data were compared to historical data (2010–2018) (Figure 2A), showing that May 2020 was particularly hotter than 2010–2019 (the recorded average temperatures were 20°C, 14°C, and 18°C in May 2020, May 2019 and May 2010–2018, respectively), while May 2019 was characterized by high daily average rainfall (7 mm) compared to May 2020 (2 mm) and the 9-year seasonal average (5 mm). These conditions favored the naturally occurring FHB in the fields of interest. In fact, in 2019, FHB severity (Figure 2B) reached 59% and 92% at ZS 65 and 71, respectively, indicating that the wet season (mean relative humidity was > 70%) was particularly favorable for the FHB to spread. Although April and May 2020 were not characterized by frequent rains (average precipitation of 2 mm), low precipitations favored a moderate FHB infection with a severity of 27% and 68% at ZS 65 and 71, respectively.

### Ground-Based Measurements During the UAV Campaigns

**FIGURE 3:**
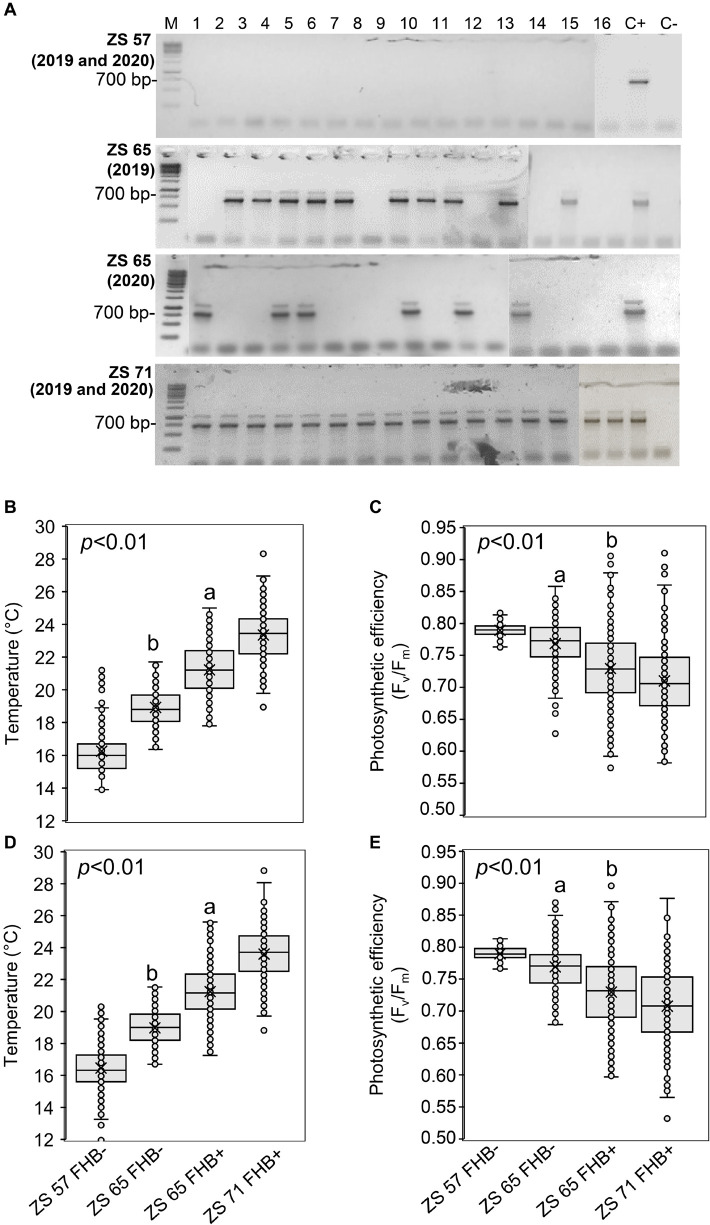
Molecular identification of Fusarium head blight (FHB) and ground-based temperature and photosynthetic efficiency measurements recorded in concurrence with unmanned aerial vehicle (UAV) campaigns conducted at Zadoks stage (ZS) 57, 65, and 71 of *Triticum turgidum* (cv. Marco Aurelio) in 2019 and 2020. **(A)** Molecular identification of *Fusarium* spp. using 1.5% agarose gel of the translational elongation factor 1-α (*TEF*) sequence (700 bp) in spikes of isolates obtained from the 16 sampling areas, sampled in 2019 and 2020 at ZS 57, 65, and 71. At ZS 57, none of the 16 sampling areas resulted FHB positive (FHB+) during both 2019 and 2020 UAV campaigns; at ZS 65, 10 and 6 sampling areas were FHB+ in 2019 and 2020, respectively; at ZS 71, all sampling areas were FHB+ during both 2019 and 2020 UAV campaigns. M represents a 100 bp DNA Ladder (Jena Bioscience); C- represents the negative control and C+ represents the presence of *F. graminearum* wild type (WT) 3824. The figure is obtained from four gels and the original pictures of the gels are available upon request. **(B,D)** Box-plot of the spikes’ temperature (°C) and **(C,E)** photosynthetic efficiency (F_v_/F_m_). The data represent averages and standard errors for 256 spikes (16 spikes for each sampling area) from 2019 **(B,C)** and 2020 **(D,E)**. Different letters refer to the statistical analysis performed using one-way analysis of variance (ANOVA) with the Tukey’s honest significant difference (HSD) post hoc test at 0.95 or 0.99 confidence level and *p* < 0.05 or *p* < 0.01.

Figure 3 illustrates the results obtained by ground-based measurements during the UAV campaigns. The molecular identification of FHB was performed by amplifying the *TEF* sequence from a bulk sample obtained from the samples collected in each sampling area, producing an amplicon of 700 bp. At ZS 57, all the sampling areas resulted FHB- in both 2019 and 2020; at ZS 65, 10 of the 16 sampling areas were FHB+ in 2019, while 6 sampling areas were FHB+ in 2020; at ZS 71, all the sampling areas were FHB+ in both 2019 and 2020 (Figure 3A). In 2019, thirty *Fusarium* morphotypes were isolated: ten morphotypes were *F. graminearum*, eleven were *F. poae*, eight were *F. avenaceum*, and one was *F. proliferatum*. In 2020, twenty-four morphotypes were identified: ten were *F. graminearum*, ten were *F. poae* and four were *F. avenaceum*. The isolated morphotypes and the data resulted from the BLASTn analyses are listed in Supplementary Table 2. Ground-based spike temperature values recorded in 2019 and 2020 revealed that FHB+ spikes had a higher temperature than the FHB- ones at ZS 65 (Figures 3B,D). On the other hand, photosynthetic efficiency had an inverse relationship with the FHB severity: variable fluorescence/maximum fluorescence (F_v_/F_m_) demonstrated to be lower in FHB+ than in FHB- at ZS 65 (Figures 3C,E).

### UAV-Based TIR and RGB Imaging for FHB Detection

**FIGURE 4:**
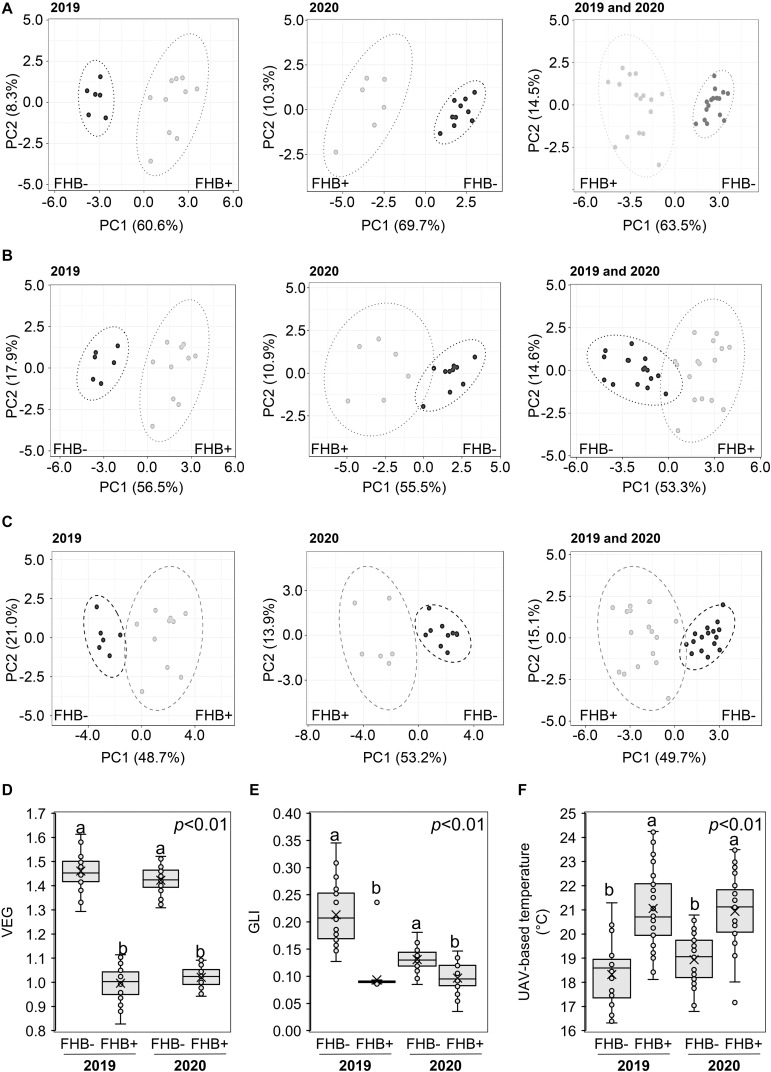
Principal component analysis (PCA) of unmanned aerial vehicle (UAV)-based **(A)** vegetative index (VEG), **(B)** green leaf index (GLI), **(C)** temperature at Zadoks stage (ZS) 65. PCA was performed by using ClustVis Software for *p* < 0.05 to distinguish between FHB+ and FHB– areas. Box-plot of UAV-based **(D)** VEG, **(E)** GLI, and **(F)** temperature from FHB– and FHB+ areas. Data were recorded during 2019 and 2020. The data represent averages and standard errors for four measurements for each sampling area from 2019 **(B,C)** and 2020 **(D,E)**. Different letters refer to the statistical analysis performed using one-way analysis of variance (ANOVA) with the Tukey’s honest significant difference (HSD) post hoc test at 0.95 or 0.99 confidence level and *p* < 0.05 or *p* < 0.01. Only the data deriving from 1 year were compared.

Recorded weather data indicated that during the two UAV-campaigns conducted at ZS 65, the average daily air temperature and humidity values were 15°C (2019), 21°C (2020), and 67% (2019) and 72% (2020), respectively. Supplementary Figure 2 shows the 16 sampling areas resulting from RGB (A and C) and thermal images (B and D) in Amelia (Supplementary Figures 2A,B) and Avigliano Umbro (Supplementary Figures 2C,D). PCA (Figure 4) demonstrated that VEG (A), GLI (B), and UAV-based temperatures (C) distinguished between FHB+ and FHB- plants at ZS 65 for both the 2019 and 2020 campaigns. Moreover, box-plots indicate that VEG (D), GLI (E), and UAV-based temperature values significantly differed between FHB+ and FHB− areas during the two UAV-campaigns (2019 and 2020). The average VEG values (D) recorded in FHB− areas were 1.46 and 1.42, while in FHB+ areas were 1.00 and 1.02 in 2019 and 2020, respectively. The average GLI values (E) recorded in FHB− areas were 0.21 and 0.13, while in FHB+ were 0.09 and 0.10 in 2019 and 2020, respectively. The average UAV-based temperature values (F) recorded in FHB− areas were 18.32°C and 18.94°C, while in FHB+ were 21.06°C and 20.94°C in 2019 and 2020, respectively.

### Monitoring of FHB in the Greenhouse

**FIGURE 5:**
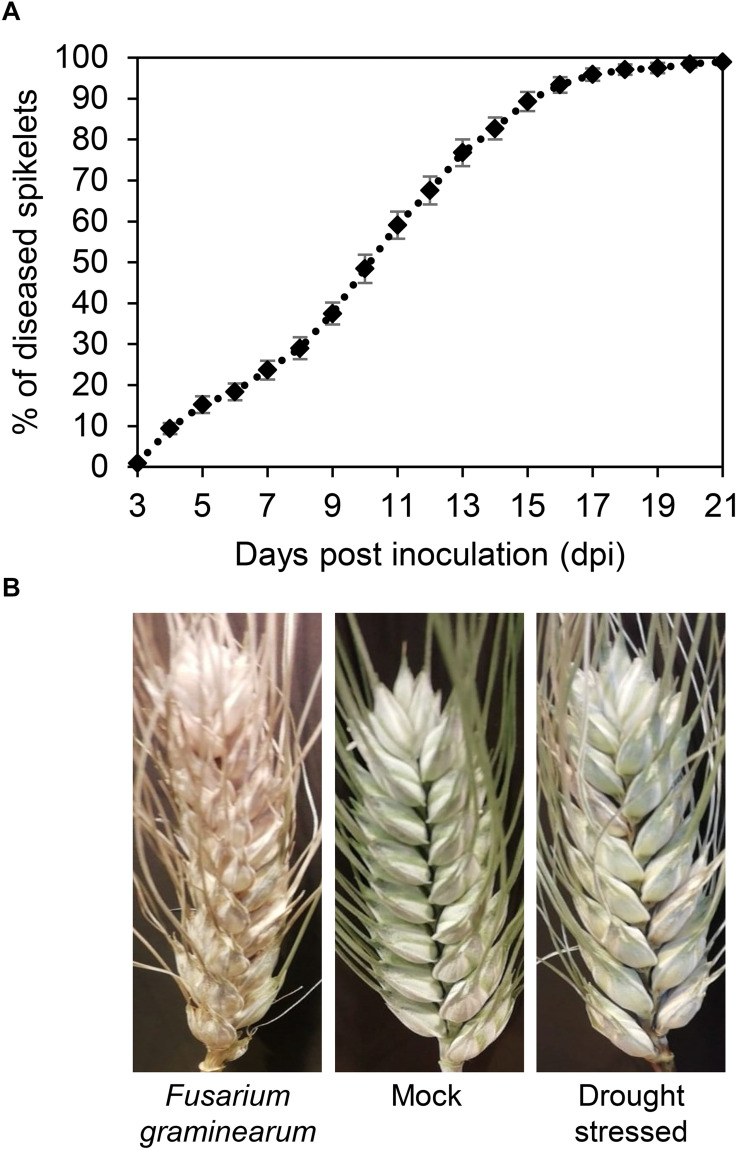
**(A)** Fusarium head blight (FHB) severity (%) in *Triticum turgidum* (cv. Marco Aurelio) from 3 to 21 days post inoculation (dpi) during the greenhouse experiments. **(B)** FHB symptoms in *T. turgidum* at 21 dpi. The data was derived from the averages and standard errors of three treatments (*F. graminearum*-inoculated, drought-stressed, and mock) with at least 20 plants for each, and three independent experiments for each treatment.

The progress of FHB severity was monitored in *F. graminearum*-inoculated plants in the greenhouse from 3 to 21 dpi (Figure 5A). The severity gradually increased reaching values close to 100% at 17 dpi, confirming the susceptibility of *T. turgidum* cv. Marco Aurelio. Figure 5B shows the FHB symptoms at 21 dpi. While no symptoms were observed on the mock treatment, few non-necrotic bleached spikelets appeared on drought-stressed plants.

### Monitoring Spike Temperature and Photosynthetic Efficiency Between Mock, Drought, and Inoculated Treatments

**FIGURE 6:**
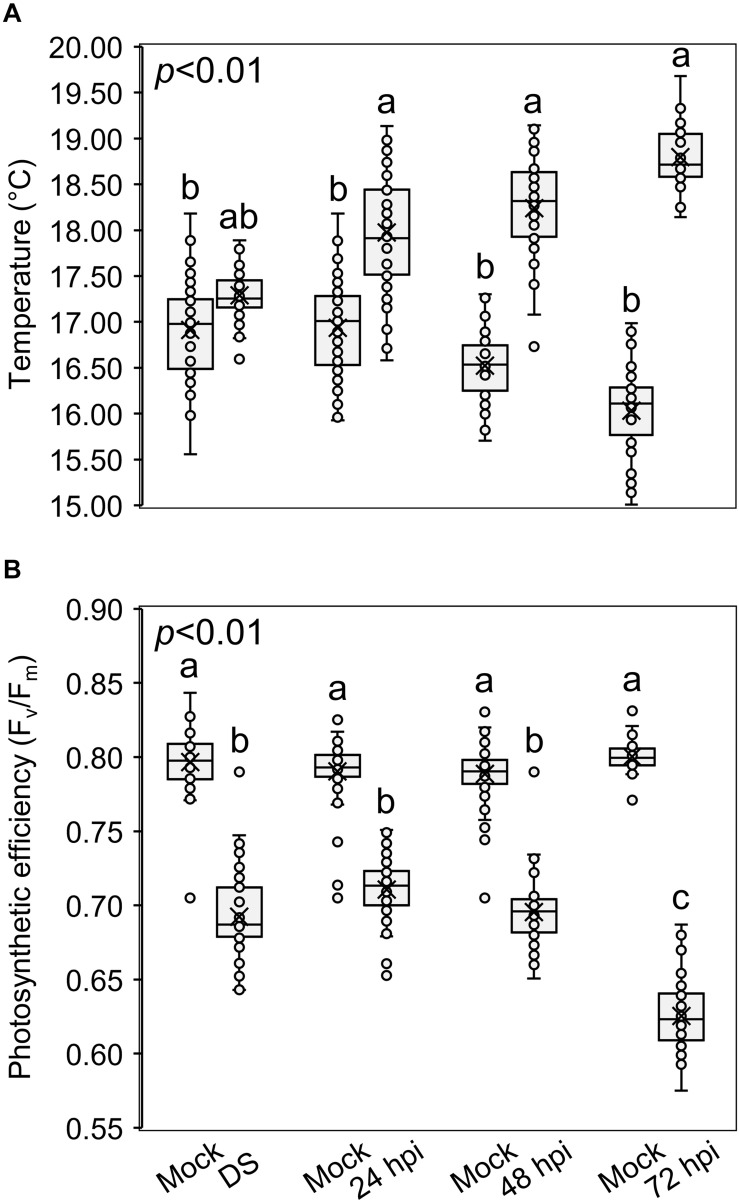
Box-plots of **(A)** spike temperatures (°C) and **(B)** photosynthetic efficiencies (F_v_/F_m_) of *Triticum turgidum* (cv. Marco Aurelio) plants subjected to mock (M), drought stress (DS) and *Fusarium graminearum* artificial inoculation (24, 48, and 72 hours post inoculation (hpi)) treatments during the greenhouse experiments. The data was derived from the averages and standard errors of three treatments (*F. graminearum*-inoculated, drought-stressed, and mock) with at least 20 plants for each, and three independent experiments for each treatment. Different letters refer to the statistical analysis performed using one-way analysis of variance (ANOVA) with the Tukey’s honest significant difference (HSD) post hoc test at 0.99 confidence level and *p* < 0.01.

Figure 6 shows the temperature of spikes (Figure 6A) and the photosynthetic efficiency (Figure 6B) after the three treatments. Compared to the mock treatment, the temperature increased and the photosynthetic efficiency decreased in drought-stressed and inoculated plants, confirming a perturbation of the photosynthetic activity. In fact, the more the infection progressed, the higher the differences in spike temperature and photosynthetic efficiency between the inoculated and mock plants: inoculated spikes reached a temperature of 17.89, 18.48, and 18.67°C while mock spikes measured 17.12, 16.44, and 16.19°C, at 24, 48 and 72 hpi, respectively; the photosynthetic efficiency measured 0.719, 0.695 and 0.618 F_v_/F_m_ for the inoculated spikes and 0.794, 0.793 and 0.801 F_v_/F_m_ for the mock at 24, 48 and 72 hpi, respectively. Notably, *F. graminearum* infection perturbated the photosynthetic parameters more than drought stress, highlighting that, by using these measures, it is possible to distinguish between drought-stressed and FHB−infected plants.

### Expression Pattern of the Genes Regulating Stomatal Conductance by RT-qPCR

E, *R*^2^, and the stability of the reference genes were calculated to validate the RT-qPCR results. E ranged from 0.9652 to 1.2741 and *R*^2^ from 0.9651 to 0.9954. Standard errors (SE) among the Cq values of the three reference genes ranged from 0.198 to 0.369 indicating their stable expression under the three different treatments.

Since metrics derived from TIR and RGB images allowed the detection of infected spikes at ZS 65, the greenhouse experiments were designed to investigate the differential stomatal regulation response in proximity of the same phenological stage.

**FIGURE 7:**
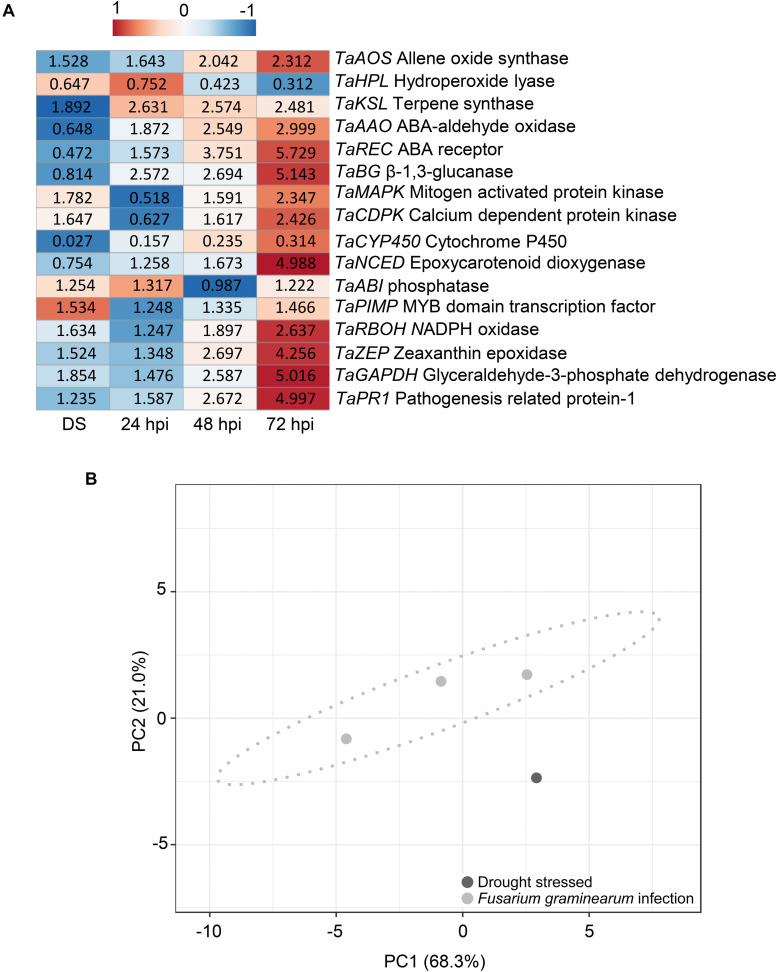
**(A)** Heatmap of relative expression level of the genes selected in *Triticum turgidum* (cv. Marco Aurelio) exposed to drought stress (DS) and *Fusarium graminearum*-inoculated at 24, 48, and 72 hours post inoculation (hpi)) treatments. The expression values were normalized to the mock treatment and to *T. aestivum* actin (*TaACT*), *T. aestivum*β-tubulin2 (*TaTUB*), and *T. aestivum* ferredoxin-NADP(H)-oxidoreductase (*TaFNR*) as reference genes. The heatmap was constructed by plotting the z-score of the relative gene expression values and it was generated by analyzing data with ClustVis Software. The red color represents the up-regulated genes, while the blue color the down-regulated genes. **(B)** Principial component analysis (PCA) of relative gene expression values, spike temperature and photosynthetic efficiency to distinguish between drought stress and *F. graminearum* infection during the greenhouse experiments. PCA was performed by using ClustVis Software for *p* < 0.05.

Moreover, drought-stressed plants were studied to observe their gene expression differences with plants under *F. graminearum* inoculation. Figure 7A shows a heatmap of the relative expression values of plant genes under drought stress and *F. graminearum* inoculation at 24, 48, and 72 hpi. Supplementary Table 3 provides the relative expression values, SE, and the HSD test computed at 0.99 confidence level. Under terminal drought stress, *T. aestivum* allene oxide synthase (*TaAOS*), *T. aestivum* terpene synthase (*TaKSL*), *T. aestivum* mitogen-activated protein kinases (*TaMAPK*), *T. aestivum* calcium dependent protein kinase (*TaCDPK*), *T. aestivum* phosphatase (*TaABI*), *T. aestivum* MYB domain transcription factor (*TaPIMP*), *T. aestivum* NADPH oxidase (*TaRBOH*), and *T. aestivum* zeaxanthin epoxidase (*TaZEP*) were slightly up-regulated showing expression values ranging from 1.254-fold to 1.892-fold. Among the different time-points of *F. graminearum* inoculation (24, 48, and 72 hpi), the relative expression values of *TaAOS, T. aestivum* abscisic acid (ABA) aldehyde oxidase (*TaAAO*), *T. aestivum* ABA receptor (*TaREC*), *T. aestivum*β-1,3-glucanase (*TaBG*), *TaMAPK, TaCDPK, T. aestivum* epoxycarotenoid dioxygenase (*TaNCED*), *TaRBOH*, and *TaZEP* gradually increased from 24 to 72 hpi, while *T. aestivum* hydroperoxide lyase (*TaHPL*) and *T. aestivum* cytochrome P450 (*TaCYP450*) were down-regulated. Notably, the expression patterns of *TaKSL, TaAAO, TaREC, TaBG, TaCYP450, TaNCED*, and *TaZEP* were different between drought-stressed and *F. graminearum-*inoculated plants. In particular, in the inoculation treatment, at 72 hpi *TaREC, TaBG, TaNCED*, and *TaZEP* were strongly up-regulated (5.729, 5.143, 4.988, and 4.256-fold change, respectively) while *TaPR1* and *TaGAPDH* were gradually up-regulated from 24 to 72 hpi, indicating that the *F. graminearum* infection perturbated the innate immunity and physiological photosynthesis of the plants. Figure 7B represents the PCA obtained by computing relative gene expression values, spike temperature and photosynthetic efficiency values from drought-stressed and *F. graminearum* inoculated plants. PCA demonstrated that these data distinguished between hydric stress and *F. graminearum* inoculation.

## Discussion

Changes in temperature and color of spikes are a result of the physiological defensive response of *T. turgidum* to FHB. Indeed, a thickening of the vascular bundles occurs when the infection moves from the floret to the rachilla which causes an increase in temperature, a decrease in photosynthesis efficiency, and a reduced transpiration due to limited water supply and stomatal closure (Kang and Buchenauer, 2000; Kheiri et al., 2019). These physiological changes allow remote sensing techniques to detect and quantify FHB in *T. turgidum* non-destructively.

Previous studies have revealed the potential of remote sensing methods in detecting and assessing plant diseases. For example, Oerke and Steiner (2010) detected FHB using thermal imaging only at a late infection stage, when it was too late for the disease to be controlled. Al Masri et al. (2017) studied the effect of the primary infection site by *F. graminearum* and *F. culmorum* using thermography under controlled conditions and they observed that FHB infection significantly increased the temperature of spikes as it progressed from 6 to 29 dpi. Mahlein et al. (2019) demonstrated that *Fusarium*-infected spikelets showed higher temperatures and lower F_v_/F_m_ values compared to mock control using a digital thermo-camera and a chlorophyll fluorometer. The authors recorded temperature and F_v_/F_m_ values in entire spikes to detect FHB infection at 5 and 7 dpi.

Red-green-blue imaging was widely employed to detect FHB-infected and FHB-damaged kernels (Jaillais et al., 2015; Cambaza et al., 2019; Abbaspour-Gilandeh et al., 2020), but few studies explored this technique to detect FHB on spikes. Huang et al. (2020) proposed an FHB diagnostic model of disease severity based on the fusion of RGB and spectral imaging. The results showed that the model was able to identify FHB severity in plants with an accuracy of 92%, thereby providing a technical basis for timely and effective control of FHB. Dammer et al. (2011) made use of RGB imaging to detect FHB in the field. Experimental plants were artificially infected with a spore suspension and RGB images were captured and analyzed to detect the disease symptoms. The authors found a linear correlation between RGB-derived and visually observed disease levels in plants. Qiu et al. (2019) accurately detected FHB in the field using RGB imaging. RGB-derived data correlated with the number of diseased spikes tallied by manual count. These results are in agreement with those obtained in our study since we observed an increase in temperature and a decrease in photosynthetic efficiency in FHB-infected spikes. Moreover, UAV-based measurements distinguished FHB+ and FHB− areas, confirming that TIR and RGB imaging are powerful tools for FHB detection. To the best of our knowledge, for FHB detection, TIR and RGB cameras have only been employed on ground-based phenotyping platforms, minimizing their portability and limiting the scale at which they can be used. Thus, this is the first study demonstrating that UAV-mounted TIR and RGB cameras enable rapid characterization of *T. turgidum* and detection of FHB in the field, overcoming the limitations associated with ground-based phenotyping.

In the present study, the relative expression level of genes involved in stomatal regulation was evaluated at ZS 65 to elucidate the genetic mechanism responsible for the phenotypic response to perturbation of photosynthesis, and to establish whether or not a differential gene response exists between drought-stressed and FHB*-*infected *T. turgidum*. Stomatal closure is the primary response of plants to water deficit, controlled by abscisic acid (ABA), a key hormone involved in controlling many aspects of plant growth, development, and responses to a variety of biotic and abiotic stresses (Daszkowska-Golec and Szarejko, 2013; Duarte et al., 2019). Our results are in agreement with the literature since the majority of stomatal closure positive regulating genes involved in ABA biosynthesis (*TaKSL, TaZEP, TaCDPK, TaMAPK, TaRBOH*, and *TaPIMP*) were induced, while the negative regulators (*TaCYP450, TaBG*, and *TaREC*) were down-regulated after being exposed to drought stress. In contrast, some positive regulators of stomatal closure (*TaNCED, TaAAO*, and *TaHPL*) were down-regulated while a negative regulator (*TaABI*) was induced. Our findings support the hypothesis stating that hydric stress conditions do not completely induce stomatal closure in drought-tolerant wheat varieties, which correlate with lower level of closure-inducing genes and higher expressions of genes negatively regulators of stomatal closure (Xue et al., 2006; Ji et al., 2011; Rampino et al., 2012; Gallé et al., 2013). Moreover, recorded temperature values of spikes in the greenhouse revealed that Marco Aurelio is moderately tolerant to drought stress since temperature measures of plants exposed to drought stress did not significantly differ from the mock. However, amongst the distinctive responses between drought-stressed and *F. graminearum*-inoculated plants, *TaAAO, TaREC, TaBG*, and *TaNCED* were down-regulated in the former and up-regulated in the latter. On the other hand, *TaMAPK* and *TaCDPK* were up-regulated in drought-stressed but not in *F. graminearum*-inoculated plants at 24 hpi. Spikes temperature and photosynthetic efficiency values of *F. graminearum-*inoculated plants differed significantly from the mock and notably, at 72 hpi, the photosynthetic efficiency allowed the distinction between *F. graminearum*-inoculated and drought-stressed plants. These observations can be extremely helpful to develop further methodologies aimed at distinguishing between drought-stressed and FHB-infected plants in the field. In this regard, biotic and abiotic stresses need to be distinguished in order to optimize practical field management. Shaik and Ramakrishna (2014) segregated biotic and abiotic stresses in rice by applying machine learning approaches to the expression levels of a set of stress-responsive genes. Focusing on the use of imaging, hyperspectral sensors are the most suitable to distinguish biotic and drought stresses in many crops (Jones, 2011; Susič et al., 2018). To date, most of the research studies distinguish between drought and disease infection applied separately, while Ramegowda and Senthil-Kumar (2015) amply reviewed experimental evidence suggesting that, under combined drought and biotic stress, plants exhibit tailored physiological and molecular responses. Such tailored responses occur only in plants exposed to simultaneous stresses and such information cannot be inferred from individual stress studies.

Additionally, in *F. graminearum*-inoculated plants, most of the positive regulators of stomatal closure (*TaAOS, TaKSL, TaAAO, TaNCED, TaPIMP, TaRBOH*, and *TaZEP*) were induced from 24 to 72 hpi while *TaMAPK* and *TaCDPK* were up-regulated at 48 and 72 hpi, confirming that early stomatal closure is the physiological mechanism behind the increasing temperature and decreasing photosynthetic efficiency in spikes. The negative stomatal closure regulators *TaBG* and *TaREC* were also remarkably up-regulated, while *TaCYP450* was down-regulated. Our results are in agreement with the literature data, reporting the induction of *TaBG* and *TaREC* and the down-regulation of *TaCYP450* in FHB-susceptible wheat cultivars. *TaBG* belongs to the pathogenesis-related proteins family (PR2) in wheat, which is known to be induced as a defense mechanism in response to biotic and abiotic stresses (Muthukrishnan et al., 2001). Particularly, De Zutter et al. (2017) investigated the *T. aestivum* response to a combined attack of *F. graminearum* and *Sitobion avenae* aphids, and observed the consistent up-regulation of *PR1* and *PR2*. Another study (Francesconi and Balestra, 2020) demonstrated that *TaPR1* and *TaPR2* were induced in an *F. graminearum-*susceptible *T. aestivum* (cv. Rebelde) but not as much as in the FHB-resistant *T. aestivum* (cv. Sumai3). The up-regulation of *TaREC* could be explained by evidence supporting that it may be involved in FHB susceptibility, since Gordon et al. (2016) found that *REC* silencing in *T. aestivum* (cv. Chinese Spring) resulted in slower progression of FHB symptoms and decreased DON content in wheat heads. On the other hand, *TaCYP450* was down-regulated in *F. graminearum-*infected *T. turgidum*. In fact, much evidence indicated that *CYP450* plays an active role in wheat resistance against FHB and DON accumulation. Strong *CYP450* accumulations were found in *F. graminearum-* and DON-resistant but not in susceptible wheat cultivars (Li et al., 2010; Gunupuru et al., 2018; Francesconi and Balestra, 2020). Several studies also demonstrated that *CYP450* was able to detoxify DON *in vitro* (Ito et al., 2013) and *in vivo* (Gunupuru et al., 2018).

The present study proved that UAV-based TIR and RGB image analysis can detect FHB infections at ZS 65. This can improve different aspects of FHB management and plant breeding. For example, our methodology allows timely detection of FHB and mapping affected locations in the field, thus optimizing the application timing and amount of fungicides needed to control the disease (Oerke and Steiner, 2010). It can also provide valuable information about the severity of FHB and help meet future food traceability requirements. Indeed, the ability to monitor FHB severity before further processing of harvested kernels can help determining whether the grains fit for human or animal consumption, with special regard to mycotoxin content (Dammer et al., 2011). For such purpose, the image-assisted analysis coupled with prediction modeling could be a valuable method to predict and detect the accumulated mycotoxin in the grains (Battilani, 2016; Leplat et al., 2018; Fernando et al., 2021). Several research studies reported also the accumulation of mycotoxin in absence of macroscopic symptoms, while microscopic analysis revealed that the host cells drastically changed after the infection (Brown et al., 2010; Peiris et al., 2011; Alisaac et al., 2021). For such reasons, the detection of mycotoxin in asymptomatic spikes could be successfully achieved by using multispectral imaging (Bauriegel et al., 2011; Dammer et al., 2011; Leplat et al., 2018) to support the mycotoxin traceability performed by the costly techniques based on chromatography (Tittlemier et al., 2021). Furthermore, the presented methodology can help quantifying host resistance to FHB in pre-breeding and commercial breeding trials (Yang et al., 2017), speeding-up breeding programs.

## Conclusion

The rapid detection of FHB is a key factor to gain maximum, environmentally sustainable protection of yield. To maximize FHB disease control efficiency, we explored the use of UAV-based RGB and TIR imaging supported by ground-truthing to detect the presence of FHB in *T. turgidum* (cv. Marco Aurelio). The present study revealed that: (i) stomatal closure is the physiological mechanism responsible for temperature increase and photosynthetic efficiency decrease in *T. turgidum* spikes during FHB infection; (ii) VIs and temperatures extracted from RGB and TIR imaging data can detect these physiological changes; and (iii) different transcriptional regulations exist between drought-stressed and *F. graminearum-*inoculated plants. These findings provide mechanisms for the detection of FHB in *T. turgidum* and shed light into new valuable genomic information to further develop a phenotyping method able to distinguish between drought-stressed and FHB-infected plants in the field.

Research in plant stress physiology is benefiting from new types of precision disease management technologies based on phenomics, genomics, and transcriptomics data. In the last decade, plant genomics and phenomics have matured to the point where, applied together, they can drastically reduce bottlenecks in phenotypic and genotypic evaluation of plant traits (Flood et al., 2011; Murchie et al., 2018; van Bezouw et al., 2019) and when coupled with artificial intelligence and exascale computing, they can accelerate the development of new crop varieties with improved yield potential and enhanced tolerance to biotic and abiotic environmental stresses (Harfouche et al., 2019; Streich et al., 2020). Their implementation and application will elucidate the architecture of plant physiological mechanisms to develop innovative tools to be applied in a new green revolution (Ray et al., 2013). To date, no studies have been carried out attempting to use UAV-based TIR and RGB imaging data for the detection of FHB in *T. turgidum*. Developing trait measurement methodologies that combine phenomics and genomics to detect plant diseases can provide a timely warning of their imminent threat, allowing decisions to be made in time for fungicides to be effective, reducing the costs and negative environmental impacts of their unnecessary applications. Further research is needed to test the reproducibility of UAV-based phenomics in different environments and to explore their potentiality to distinguish between biotic and abiotic stresses.

## Data Availability Statement

The original contributions presented in the study are included in the article/Supplementary Material, further inquiries can be directed to the corresponding authors.

## Author Contributions

SF, AH, MM, and GB conceived and designed the experiments. SF, AH, and GB led the writing of the manuscript and figure preparation, and contributed to the majority of the critical revision of the manuscript text and figures. SF led the ground-based experiments, data collection, and analysis. MM led the UAV-based experiment, data collection, and analysis. All authors have revised the work for intellectual content and have contributed to and approved the final content.

## Conflict of Interest

The authors declare that the research was conducted in the absence of any commercial or financial relationships that could be construed as a potential conflict of interest.

## Footnotes

1 https://annali.regione.umbria.it/

2 https://blast.ncbi.nlm.nih.gov/

3 https://biit.cs.ut.ee/clustvis/
