# Supplementary material for: UAV-Based Thermal, RGB Imaging and Gene Expression Analysis Allowed Detection of Fusarium Head Blight and Gave New Insights Into the Physiological Responses to the Disease in Durum Wheat
Source: Front Plant Sci. 2021 Apr 1;12:628575. doi: 10.3389/fpls.2021.628575 (PMC8047627; doi:10.3389/fpls.2021.628575)
Supplement: Supplementary file 1 [file Data_Sheet_1.PDF]

Supplementary Material

1 Supplementary Figures and Tables

1.1 Supplementary Figures

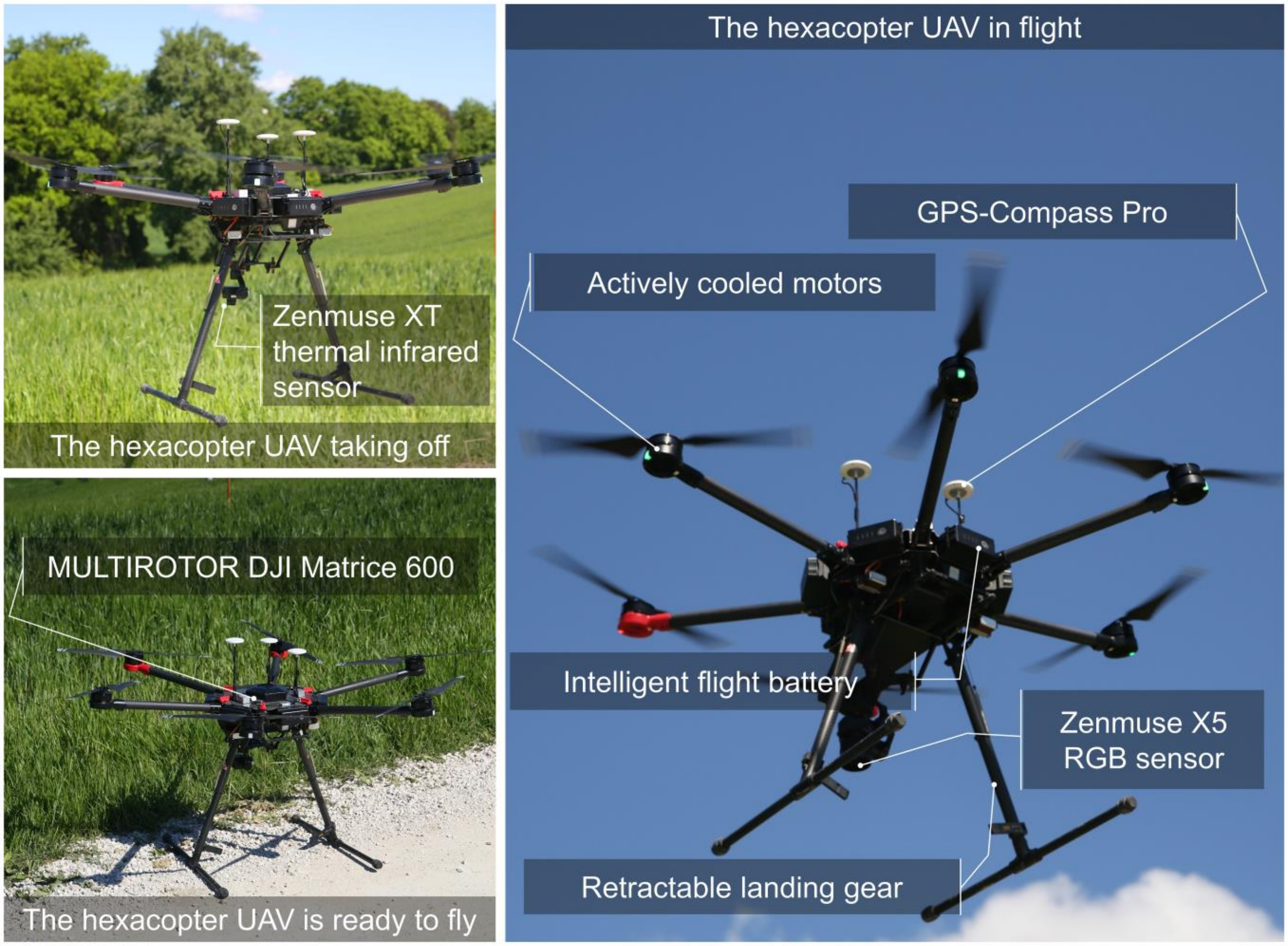

Figure S1. The DJI Matrice 600 unmanned aerial vehicle (UAV) platform used for field phenotyping of *Triticum turgidum* (cv. Marco Aurelio) on the ground, taking off, and in flight.

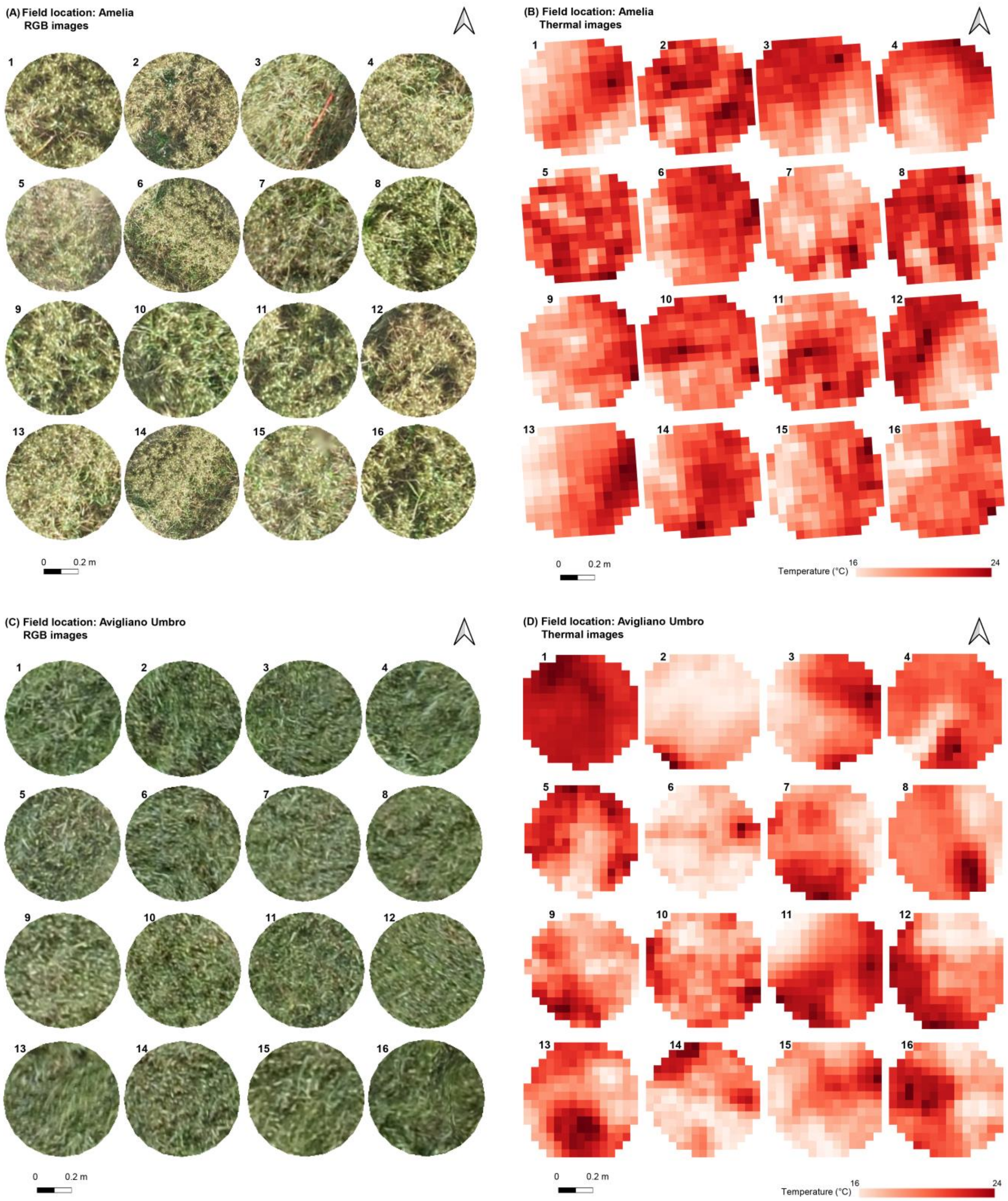

Figure S2. (A, C) Red-green-blue (RGB) and **(B, D)** thermal images of the 16 sampling areas in Amelia and Avigliano Umbro field sites, respectively. Images were calibrated and orthomosaicked using the Pix4Dmapper software (Pix4D, Switzerland). FHB+ areas had an average temperature of around 21°C while FHB− areas had an average temperature of around 19°C.

1.2 Supplementary Tables

Table S1. List of target genes, accession numbers, gene functions, and primer pairs. The forward (F) and reverse (R) primers used for amplifications of *Triticum aestivum* genes: primers for *TaGAPDH* are from Jarošová and Kundu (2010), for *TaPR1* from Lu et al. (2006), for *TaACT* from Tundo et al. (2016), and for *TaTUB* and *TaFNR* from Tenea et al. (2011). The remaining primers are from Francesconi and Balestra (2020).

Gene and Accession Number	Function	Primer name	Primer pairs (5'-3')
TaAOS AY196004	Allene oxide synthase	TaAOS_F<br>TaAOS_R	TCGGGCGTATTGCTGAGG<br>TGCAGCAGCTTGCTTCTCTC
TaHPL AK335301	Hydroperoxide lyase	TaHPL_F<br>TaHPL_R	GGACCACAGCATGACCGACA<br>GAACCCGACCTCGCCGTT
TaKSL AB597957	Terpene synthase	TaKSL_F<br>TaKSL_R	GGAACGGGATGCTAGAATACG<br>CCTCTCCTTATGTGGTCGGA
TaAAO AK331622	ABA-aldehyde oxidase	TaAAO_F<br>TaAAO_R	CCTGAGCAATCAAAGCATCCC<br>TCAGGCTGACACGCTGAACT
TaREC AK335719	ABA receptor	TaREC_F<br>TaREC_R	GCTGGAGATCCTGGACGAC<br>GTTGCACTTGACGATGGTGT
TaBG Y18212	β-1,3-glucanase	TaBG_F<br>TaBG_R	AACGTGCGCCCCTACTACC<br>GCGTCGAACAGGCTCGTGTA
TaMAPK AF079318	Mitogen activated protein kinase	TaMAPK_F<br>TaMAPK_R	CATCGACGTCTGGTCCGT<br>GTCCTCGTTCCGGATGAATC
TaCDPK KU516994	Calcium dependent protein kinase	TaCDPK_F<br>TaCDPK_R	CTTCTTTGTGGTGTCCCTCC<br>GCTGTCAAACGCCTCCTT
TaCYP450 XM_020311851	Cytochrome P450	TaCYP_F<br>TaCYP_R	GTATTGGTGGACGAGGAAGG<br>ATCTGGCGTGCGATCACT
TaNCED KX711891	Epoxycarotenoid dioxygenase	TaNCED_F<br>TaNCED_R	CGGTGGAGAGGCAGGAGAA<br>CGCGCGTAGAACAGAGCA
TaABI AB238930	Phosphatase	TaABI_F<br>TaABI_R	TAGCAGATTACTGTCGGGATCGG<br>GAGCAGATGACAGCGACCA
TaPIMP KX683396	MYB domain transcription factor	TaPIMP_F<br>TaPIMP_R	GTCACAGATCGCGTCGCAC<br>GCATCCGAACTGGCCGTACA
TaRBOH AY561153	NADPH oxidase	TaRBOH_F<br>TaRBOH_R	TTGTTGGATTAGGAATTGGTGCT<br>TGATCCATGTCGGCAATCTC
TaZEP AF384103	Zeaxanthin epoxidase	TaZEP_F<br>TaZEP_R	CTTGTCACCAGCCACTGTAC<br>GGCACAACAATGTACTGTACTAGG
TaGAPDH KR029493.1	Glyceraldehyde-3-phosphate dehydrogenase	TaGAPDH_F<br>TaGAPDH_R	AGTTCATGCCATGACTGCAA<br>CCAGTGCTGCTTGGAATGATG
TaPR1 AJ007348	Pathogenesis related protein-1	TaPR1_F<br>TaPR1_R	ACTACGACTACGGGTCCAACA<br>TCGTAGTTGCAGGTGATGAAG
TaACT AB181991	Actin	TaACT_77F<br>TaACT_312R	TCCTGTGTTGCTGACTGAGG<br>GGTCCAAACGAAGGATAGCA
TaTUB TAU76745	β-tubulin2	TaTUB_F<br>TaTUB_R	CGAGGAGGGCGAGTACGA<br>AGCAAAGCACGACATGGACAT
TaFNR AJ457980	Ferredoxin - NADP(H)-oxidoreductase	TaFNR_F<br>TaFNR_R	CACCGGCCCAGTGATCTT<br>AAGGGCGTCTGCTCCAACT

Table S2. List of *Fusarium* morphotypes isolated during the two UAV campaigns (2019 and 2020). The code indicates the numerical code of the morphotype (1-30) and the year (2019 or 2020) indicates the year of isolation. The table represents the data derived from the BLASTn (https://blast.ncbi.nlm.nih.gov/) analyses.

Code	Scientific name	Max score	Total score	Query cover	E value	Percentage of identity	Accession Lenght	Accession
1_2019	*F. graminearum*	1201	1201	99%	0	99.85%	658	MK507898.1
2_2019	*F. graminearum*	1201	1201	99%	0	99.85%	658	MK507898.1
3_2019	*F. graminearum*	1201	1201	99%	0	99.85%	658	MK507898.1
4_2019	*F. graminearum*	1201	1201	99%	0	99.85%	658	MK507898.1
5_2019	*F. graminearum*	1201	1201	99%	0	99.85%	658	MK507898.1
6_2019	*F. graminearum*	1201	1201	99%	0	99.85%	658	MK507898.1
7_2019	*F. graminearum*	1201	1201	99%	0	99.85%	658	MK507898.1
8_2019	*F. graminearum*	717	717	97%	0	100%	658	HQ702570.1
9_2019	*F. graminearum*	702	702	96%	0	100%	658	HQ702570.1
10_2019	*F. graminearum*	704	704	97%	0	100%	658	HQ702570.1
11_2019	*F. poae*	1177	1177	99%	0	99.69%	644	KJ947335.1
12_2019	*F. poae*	1177	1177	99%	0	99.69%	644	KJ947335.1
13_2019	*F. poae*	1177	1177	99%	0	99.69%	644	KJ947335.1
14_2019	*F. poae*	1177	1177	99%	0	99.69%	644	KJ947335.1
15_2019	*F. poae*	1177	1177	99%	0	99.69%	644	KJ947335.1
16_2019	*F. poae*	1177	1177	99%	0	99.69%	644	KJ947335.1
17_2019	*F. poae*	1177	1177	99%	0	99.69%	644	KJ947335.1
18_2019	*F. poae*	1177	1177	99%	0	99.69%	644	KJ947335.1
19_2019	*F. poae*	1151	1151	98%	0	100%	658	HE802673.1
20_2019	*F. poae*	1151	1151	98%	0	100%	658	HE802673.1
21_2019	*F. poae*	1151	1151	98%	0	100%	658	HE802673.1
22_2019	*F. avenaceum*	1199	1199	98%	0	99.85%	727	MK577935.1
23_2019	*F. avenaceum*	1199	1199	98%	0	99.85%	727	MK577935.1
24_2019	*F. avenaceum*	1199	1199	98%	0	99.85%	727	MK577935.1
25_2019	*F. avenaceum*	1199	1199	98%	0	99.85%	727	MK577935.1
26_2019	*F. avenaceum*	1199	1199	98%	0	99.85%	727	MK577935.1
27_2019	*F. avenaceum*	1199	1199	98%	0	99.85%	727	MK577935.1
28_2019	*F. avenaceum*	1199	1199	98%	0	99.85%	727	MK577935.1
29_2019	*F. avenaceum*	1199	1199	98%	0	99.85%	727	MK577935.1
30_2019	*F. proliferatum*	1182	1182	98%	0	99.69%	703	MK952799.1
1_2020	*F. graminearum*	1201	1201	99%	0	99.85%	658	MK507898.1
2_2020	*F. graminearum*	1201	1201	99%	0	99.85%	658	MK507898.1
3_2020	*F. graminearum*	1201	1201	99%	0	99.85%	658	MK507898.1
4_2020	*F. graminearum*	1201	1201	99%	0	99.85%	658	MK507898.1
5_2020	*F. graminearum*	1201	1201	99%	0	99.85%	658	MK507898.1
6_2020	*F. graminearum*	1201	1201	99%	0	99.85%	658	MK507898.1
7_2020	*F. graminearum*	1201	1201	99%	0	99.85%	658	MK507898.1

8_2020	*F. graminearum*	1201	1201	99%	0	99.85%	658	MK507898.1
9_2020	*F. graminearum*	1201	1201	99%	0	99.85%	658	MK507898.1
10_2020	*F. graminearum*	1201	1201	99%	0	99.85%	658	MK507898.1
11_2020	*F. poae*	1153	1153	98%	0	100%	627	MH582306.1
12_2020	*F. poae*	1153	1153	98%	0	100%	627	MH582306.1
13_2020	*F. poae*	1153	1153	98%	0	100%	627	MH582306.1
14_2020	*F. poae*	1153	1153	98%	0	100%	627	MH582306.1
15_2020	*F. poae*	1153	1153	98%	0	100%	627	MH582306.1
16_2020	*F. poae*	1153	1153	98%	0	100%	627	MH582306.1
17_2020	*F. poae*	1146	1146	99%	0	99.52%	664	MK629641.1
18_2020	*F. poae*	1146	1146	99%	0	99.52%	664	MK629641.1
19_2020	*F. poae*	1146	1146	99%	0	99.52%	664	MK629641.1
20_2020	*F. poae*	1146	1146	99%	0	99.52%	664	MK629641.1
21_2020	*F. avenaceum*	1199	1199	98%	0	99.85%	684	JX397840.1
22_2020	*F. avenaceum*	1199	1199	98%	0	99.85%	684	JX397840.1
23_2020	*F. avenaceum*	1199	1199	98%	0	99.85%	684	JX397840.1
24_2020	*F. avenaceum*	1199	1199	98%	0	99.85%	684	JX397840.1

Table S3. Relative expression levels and standard errors of each selected gene for every plant treatment (drought stress, 24 hours post inoculation (hpi), 48 hpi and 72 hpi) for *Triticum turgidum* (cv. Marco Aurelio). Relative expression values were obtained using the equation $2^{-\Delta\Delta Cq}$, where ΔCq is the difference between the quantification cycles (Cq) of the target and reference genes, and ΔΔCq is the difference between ΔCq of the treatment and of the mock, with *TaACT*, *TaTUB*, and *TaFNR* as *Triticum aestivum* reference genes. The mock treatment was used to normalize the relative expression levels. Data represent averages and standard errors for the four independent biological replicates and the four technical replicates examined. Letters (a–d) represent different levels of statistical significance obtained by performing one-way analysis of variance (ANOVA) with the Tukey test at 0.99 confidence level and $p<0.01$.

Gene	Function		Drought stress	24 hpi	48 hpi	72 hpi
TaAOS	Allene oxide synthase	Relative expression	1.528	1.643	2.042	2.312
		Standard error	0.052	0.048	0.053	0.030
		$p<0.01$	b	b	a	a
TaHPL	Hydroperoxide lyase	Relative expression	0.647	0.752	0.423	0.312
		Standard error	0.023	0.015	0.018	0.022
		$p<0.01$	a	a	b	b
TaKSL	Terpene synthase	Relative expression	1.892	2.631	2.574	2.481
		Standard error	0.069	0.099	0.047	0.044
		$p<0.01$	b	a	a	a
TaAAO	ABA-aldehyde oxidase	Relative expression	0.648	1.872	2.549	2.999
		Standard error	0.002	0.014	0.039	0.047
		$p<0.01$	b	a	a	a
TaREC	ABA receptor	Relative expression	0.472	1.573	3.751	5.729
		Standard error	0.067	0.150	0.238	0.275
		$p<0.01$	d	c	b	a
TaBG	β-1,3-glucanase	Relative expression	0.814	2.572	2.694	5.143
		Standard error	0.048	0.123	0.247	0.367
		$p<0.01$	c	b	b	a
TaMAPK	Mitogen activated protein kinase	Relative expression	1.782	0.518	1.591	2.347
		Standard error	0.150	0.027	0.268	0.254
		$p<0.01$	b	c	b	a
TaCDPK	Calcium dependent protein kinase	Relative expression	1.647	0.627	1.617	2.426
		Standard error	0.255	0.046	0.990	0.271
		$p<0.01$	b	c	b	a
TaCYP450	Cytochrome P450	Relative expression	0.027	0.157	0.235	0.314
		Standard error	0.003	0.014	0.036	0.085
		$p<0.01$	b	a	a	a
TaNCED	Epoxycarotenoid dioxygenase	Relative expression	0.754	1.258	1.673	4.988
		Standard error	0.065	0.185	0.214	0.257
		$p<0.01$	c	b	b	a
TaABI	Phosphatase	Relative expression	1.254	1.317	0.987	1.222
		Standard error	0.014	0.032	0.042	0.074
		$p<0.01$	a	a	a	a
TaPIMP	MYB domain transcription factor	Relative expression	1.534	1.248	1.335	1.466
		Standard error	0.124	0.233	0.241	0.187
		$p<0.01$	a	a	a	a
TaRBOH	NADPH oxidase	Relative expression	1.634	1.247	1.897	2.637
		Standard error	0.142	0.255	0.144	0.241
		$p<0.01$	b	c	b	a
TaZEP	Zeaxanthin epoxidase	Relative expression	1.524	1.348	2.697	4.256

		Standard error	0.245	0.314	0.125	0.524
		p<0.01	c	c	b	a
TaGAPDH	Glyceraldehyde-3-phosphate dehydrogenase	Relative expression	1.854	1.476	2.587	5.016
		Standard error	0.124	0.314	0.417	0.237
		p<0.01	c	c	b	a
TaPR1	Pathogenesis related protein-1	Relative expression	1.235	1.587	2.672	4.997
		Standard error	0.185	0.236	0.114	0.255
		p<0.01	c	c	b	a

2 References

Francesconi, S., and Balestra, G. M. (2020). The modulation of stomatal conductance and photosynthetic parameters is involved in Fusarium head blight resistance in wheat. *PLoS One* 15, e0235482. doi:10.1371/journal.pone.0235482.

Jarošová, J., and Kundu, J. K. (2010). Validation of reference genes as internal control for studying viral infections in cereals by quantitative real-time RT-PCR. *BMC Plant Biol.* 10, 1–9. doi:10.1186/1471-2229-10-146.

Lu, Z. X., Gaudet, D., Puchalski, B., Despins, T., Frick, M., and Laroche, A. (2006). Inducers of resistance reduce common bunt infection in wheat seedlings while differentially regulating defence-gene expression. *Physiol. Mol. Plant Pathol.* 67, 138–148. doi:10.1016/j.pmpp.2005.12.001.

Tenea, G. N., Peres Bota, A., Cordeiro Raposo, F., and Maquet, A. (2011). Reference genes for gene expression studies in wheat flag leaves grown under different farming conditions. *BMC Res. Notes* 4, 373. doi:10.1186/1756-0500-4-373.

Tundo, S., Janni, M., Moscetti, I., Mandalá, G., Savatin, D., Blechl, A., et al. (2016). PvPGIP2 accumulation in specific floral tissues but not in the endosperm limits *Fusarium graminearum* infection in wheat. *Mol. Plant-Microbe Interact.* 29, 815–821. doi:10.1094/MPMI-07-16-0148-R.
